# Systemic Capillary Responses to Acute Exercise in Hypertensive Seniors: Insights from a Single-Center Pilot Study

**DOI:** 10.3390/jcm13102818

**Published:** 2024-05-10

**Authors:** Misa Miura, Masahiro Kohzuki, Chie Saito, Satoshi Sakai, Hisashi Sugaya, Shingo Koyama, Yasushi Matsui, Tohru Sakuma, Osamu Ito, Kunihiro Yamagata

**Affiliations:** 1 Faculty of Health Sciences, Tsukuba University of Technology, Kasuga 4-12-7, Tsukuba 305-8521, Japan; ssakai@k.tsukuba-tech.ac.jp (S.S.); h.sugaya@k.tsukuba-tech.ac.jp (H.S.); s.koyama@k.tsukuba-tech.ac.jp (S.K.); matsui@k.tsukuba-tech.ac.jp (Y.M.); sakuma@k.tsukuba-tech.ac.jp (T.S.); 2 Yamagata Prefectural University of Health Sciences, 260 Kamiyanagi, Yamagata 990-2212, Japan; makohzuki@gmail.com; 3 Department of Nephrology, Faculty of Medicine, University of Tsukuba, Tsukuba 305-8577, Japan; chie.saito@md.tsukuba.ac.jp (C.S.); k-yamaga@md.tsukuba.ac.jp (K.Y.); 4 Division of General Medicine and Rehabilitation, Faculty of Medicine, Tohoku Medical Pharmaceutical University, Sendai 981-8558, Japan; oito@hosp.tohoku-mpu.ac.jp

**Keywords:** nailfold capillary, acute exercise, hypertensive elderly, health-related parameters

## Abstract

**Objective:** The aim of this study was to investigate nailfold capillary parameters in community-dwelling individuals aged over 60 years who have hypertension and do not exercise regularly. Furthermore, the study examined the correlations between capillary function and other health-related indicators. Design: This study was a single- center pilot trial. Setting: The study took place in the Faculty of Health, Tsukuba University of Technology, Japan. Participants: Hypertensive community-dwelling elderly people took part in the study. Intervention: Microcirculation was observed before and 1 min after an arm-curl exercise by means of capillary microscopy of the non-exercised limb. Additionally, we examined other health-related indicators. **Methods**: We measured the acute effects of reperfusion on nailfold density, flow, and diameters. Secondary outcomes included the correlations between microvascular parameters and other health-related indicators. We hypothesized that brief exercise could enhance microcirculation reperfusion and correlate with other health-related parameters. **Results:** There were 20 participants with a mean (SD) age of 67.1 (5.8) years. The capillary flow rate changed from 2.3 ± 6.7 to 2.7 ± 0.2 log µm/s (*p* < 0.01), and the capillary density changed from 0.8 ± 0.2 to 0.9 ± 0.1 log/mm (*p* < 0.01), which included a significant increase in the non-exercising limb. Significant correlations were observed between the nailfold capillary diameter and body fat mass, the capillary diameter and physical activity, and the capillary density and bone mineral density. **Conclusions:** The acute effects of exercise on high-risk elderly individuals can be safe, and even 1 of min exercise can potentially improve their nailfold capillary function, despite the brief time, compared to no exercise. The results indicate that capillaries have an impact on the function of the whole body. Thus, they may be a useful diagnostic tool for assessing nailfold capillaries.

## 1. Introduction

Hypertension is a global public health issue and a major cause of morbidity and mortality. Because of population growth and aging, the number of people with uncontrolled hypertension rose from 600 million in 1980 to nearly 1 billion in 2008 [1]. In the global population, the prevalence of hypertension is 26.4%, which is expected to increase to one-third of the population by 2025 [2]. Functional and structural modifications in microcirculation occur early in hypertension development and in aging [3,4,5]. Previous studies have demonstrated that a reduction in dysfunctional vessels at rest eventually leads to anatomical micro vessel loss (structural rarefaction) as well as the functional impairment of capillary perfusion and recruitment [3,4], which are referred to as “ghost vessels” or “dysfunctional vessels”. Microcirculation in the nailfold is considered to be a predictive and noninvasive marker of generalized microvascular and endothelial function [6]. The restoration of microvascular function can help decrease systemic blood pressure by reducing peripheral vascular resistance; this, in turn, can improve circulation in target organs and help decrease cardiovascular disease complications [7]. Microvascular rarefaction appears to be an early vascular structural alteration in the setting of hypertension, as it is already present in individuals presenting with borderline hypertension and normotensive young adults with a familial predisposition to high blood pressure. The chronic increases in blood pressure that occur during senescence secondary to microcirculatory changes induce vasoconstriction within microcirculation, which promotes the development of tissue hypoxia and reduces both arteriolar and capillary density. This phenomenon contributes to additional increases in peripheral vascular resistance and establishes a vicious cycle that culminates in both tissue injury and target organ damage, which are equally present in senescence and hypertension [8]. Furthermore, elderly people with high lean mass and low-fat mass exhibit the best arterial and bone profiles, characterized by the lowest arterial stiffness and highest bone density [9].

On the other hand, pharmacotherapy and exercise are effective in improving capillary function [10,11] and other health-related indicators [12]. However, to date, the impact of local exercise on the circulation in the non-exercising limbs remains unknown. Furthermore, details of the immediate impact of exercise on the peripheral circulation in middle-aged and elderly patients with hypertension who are at a high risk of adverse events are lacking. One of the adverse effects of exercise is the rapid activation of the sympathetic nervous system at the start of exercise [13,14], which significantly affects the peripheral circulation [15]. Therefore, it is important to ensure the safety of exercise for high-risk individuals. For this reason, the present study aimed to investigate nailfold capillary parameters in community-dwelling individuals aged over 60 years who have hypertension and do not exercise regularly. Specifically, microcirculation was observed before and 1 min after an arm-curl exercise by means capillary microscopy of the non-exercised limb. Furthermore, the study examined the correlations between capillary function and other health-related indicators. 

## 2. Materials and Methods

### 2.1. Participants

**Figure 1:**
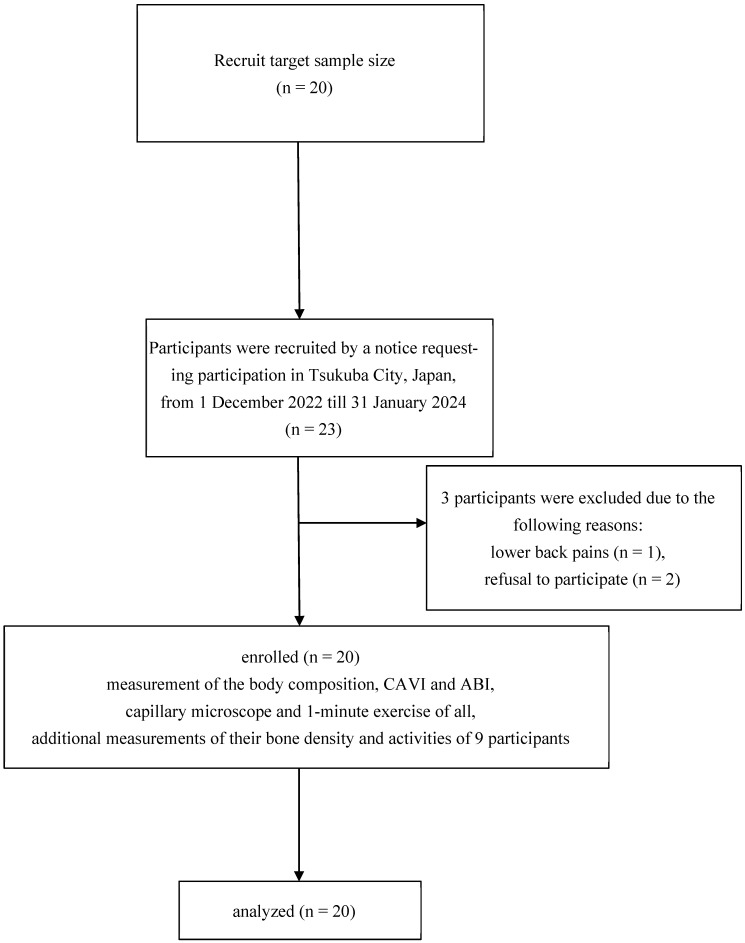
Flow chart showing enrollment.

This study enrolled community-dwelling elderly individuals with hypertension who attended public facilities in Tsukuba City, Japan, between December 2022 and January 2024 (Figure 1). Of the 23 eligible participants, 20 provided informed consent. The baseline characteristics of the participants are shown in Table 1. The inclusion criteria for the study are as follows: (1) community-dwelling elderly individuals aged over 60 who do not have a regular exercise habit, (2) individuals who met the criteria for hypertension at the time of measurement [16], (3) individuals who were briefed about the study and gave their consent to participate. The exclusion criteria were as follows: (1) patients with systolic blood pressure of 180 mmHg or higher or diastolic blood pressure of 110 mmHg or higher; (2) individuals requiring acute treatment for acute coronary syndrome, unstable angina, or other conditions; (3) individuals deemed ineligible for the study by the investigator for other reasons and those with contraindications to exercise therapy as described in the American College of Sports Medicine’s Guidelines for Exercise Testing and Prescription, 9th edition [17]. The ethics committee of Tsukuba University of Technology approved the research protocol (approval number: 202205). Furthermore, the study was registered with the University Hospital Medical Network Clinical Trials Registry (Identifier UMIN000053790). 

### 2.2. Study Design

The study followed a cross-sectional, single-center pilot trial design.

**Figure 2:**
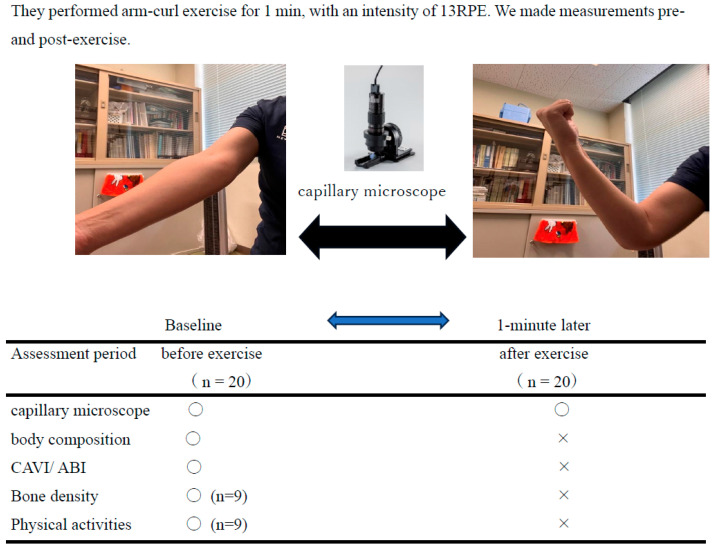
Procedure and measurement set.

The study protocol is depicted in Figure 2. The body composition and arterial stiffness of the participants were measured. Then, the participants were asked to perform elbow flexion and extension exercises in a sitting position, with the exercise intensity set at or below 13 on the RPE scale for 1 min. The arm-curl exercise and other measurements were performed with attention to the safety of the participants, under the supervision of cardiologists and orthopedic surgeons, in accordance with the exercise cessation criteria for hypertensive patients. Before and after the exercise, the capillary diameter and the flow at the nailfold of the fourth finger for the non-exercising limb were measured with a microscope.

During the session, the temperature was maintained at 23 °C. All experiments were conducted simultaneously between 9 and 11 a.m. The participants were prohibited from engaging in vigorous exercise, drinking caffeinated beverages, and smoking within 48 h before the experiment.

### 2.3. Sample Size

The sample size was calculated using G*power ver. 3.9.1.7 (Heinrich-Heine-University Dusseldorf, North Rhine-Westphalia, Germany). The target number of participants was 20 (α = 0.05, 1 − β = 0.95), which was based on a study in which capillary examination was conducted in elderly patients [18,19].

### 2.4. Measurement of Nailfold Capillary Parameters

**Figure 3:**
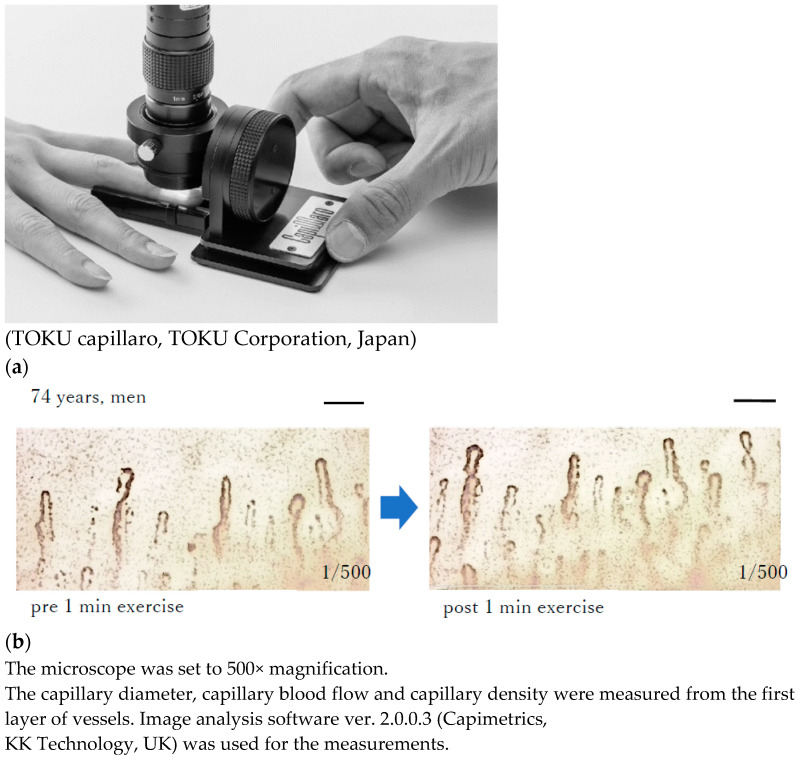
(**a**) Measurement of the nailfold parameters; (**b**) Nailfold capillary image of pre and post 1 min exercise of typical subject by the microscope measurement.

Capillary flow, diameter, and density were measured using a microscope (TOKU capillaro, Toku Corporation, Tokyo, Japan) by filming the nailfold of the nondominant fourth digits of the subjects [19,20]. Image analysis software ver. 2.0.0.3 (Capimetrics, KK Technology, Honiton, UK) was used for the measurements. During filming, the magnification of the blood flow scope was set to 500× (width, 0.87 mm; height, 0.65 mm; depth, 0.3 mm), and 10 s videos of the nailfold of the non-exercising limb were taken [20]; furthermore, the capillary diameter, blood flow, and density were measured from the first layer of the vessels, as shown in Figure 3a,b.

### 2.5. Body Composition and Physical Activities

The participants’ weight, skeletal muscle mass, and fat mass were measured by a physical therapist using a body composition analyzer MC-780A (Tanita Corporation, Tokyo, Japan). In addition, their height and physical activities were read from their application forms [21]. 

### 2.6. Measurement of the CAVI and ABI

The ankle–brachial index (ABI) and cardio-ankle vascular index (CAVI) of the participants were measured in a supine position in an air-conditioned room using a VS-1000 instrument (Fukuda Denshi, Tokyo, Japan) [22]. All subjects were instructed to lie in a supine position, with their heads held at the midline position and the palms of their hands turned upwards from their sides. After a resting period of 15 min, the measurements were taken. Electrocardiograph electrodes were placed on both wrists to collect the ECG waveform. A minitype recorder for detecting heart sounds was positioned over the fourth rib at the left edge of the sternum, and blood pressure cuffs were wrapped around both arms and ankles. The instruments automatically measured the CAVI and ABI values, and the data were subsequently analyzed computationally. 

### 2.7. Bone Mineral Density

The bone mineral density (BMD) was measured at the right heel while the subject was in a seated position, using the Benus evo instrument (Nihon Kohden Co., Tokyo, Japan) [23]. The device was calibrated daily as per the manufacturer’s recommendation. All measurements were conducted by the same operator. This bone densitometer employs both ultrasonic pulse reflection and ultrasonic pulse transmission methods to measure the sound velocity of the calcaneus alone. This is achieved by measuring the reflection distance to the bone, thereby excluding the fleshy material surrounding the calcaneus.

### 2.8. Statistical Analysis

The baseline characteristics were expressed as mean ± standard deviation. All statistical analyses were conducted using the SPSS software ver. 21 (IBM Corp, Chicago, IL, USA). Student’s *t*-test was employed to statistically analyze the logarithmically transformed wear data, and Pearson’s product-moment correlation coefficient was used for parameters of capillaries and other parameters.

## 3. Results

**Figure 4:**
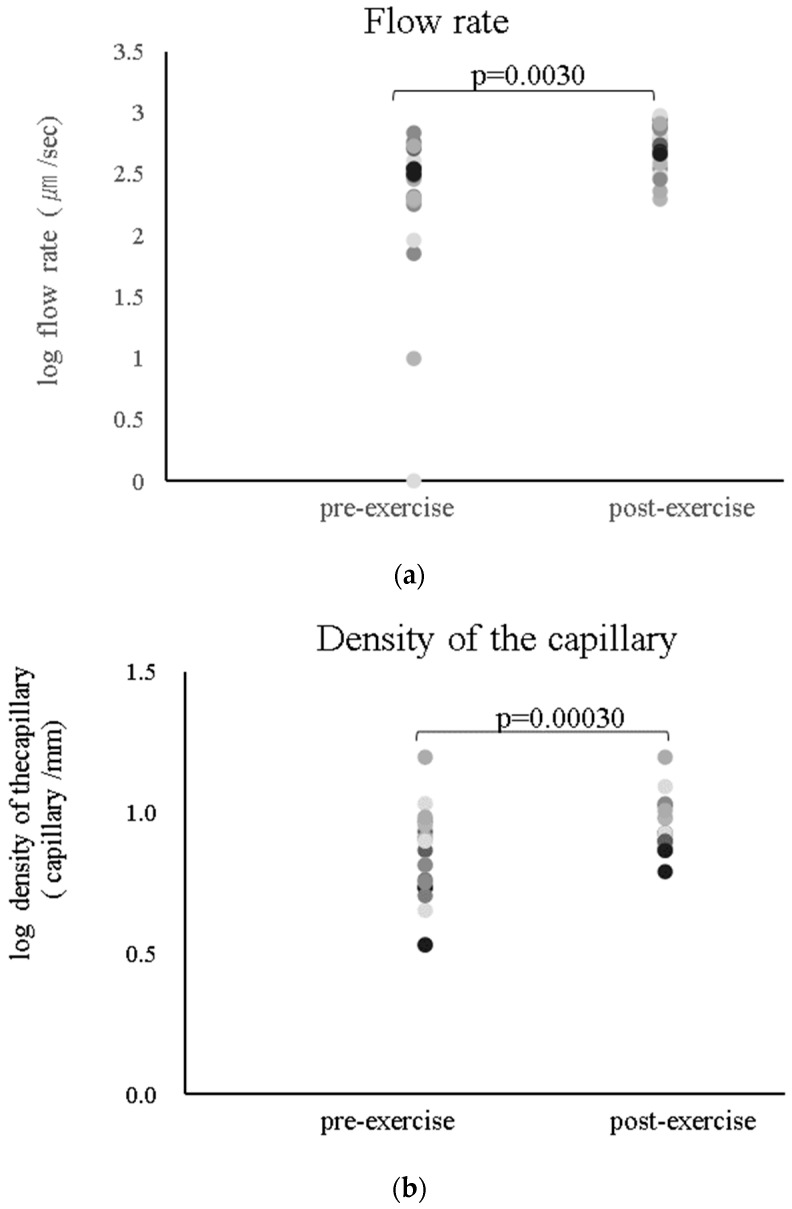
(**a**) Change in log flow rate; (**b**) change in the capillary density.

**Figure 5:**
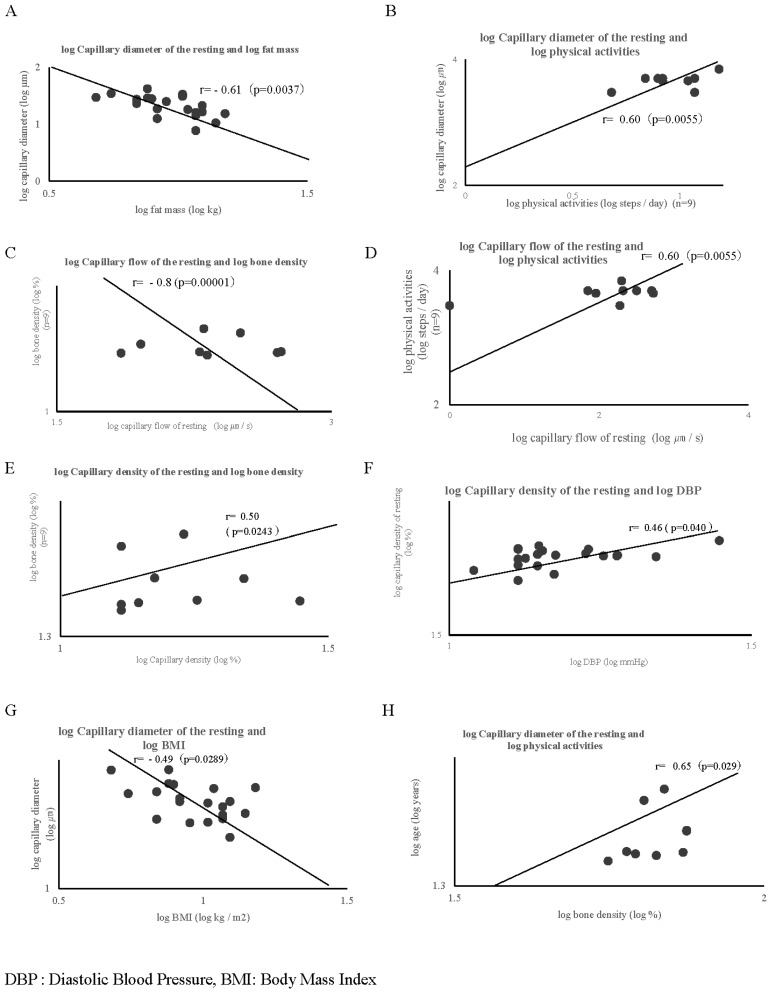
Correlation of nailfold capillary parameters at rest with other health-related parameters. (**A**) shows that the log capillary diameter at rest is significantly correlated with the log fat mass (r = −0.61; *p* = 0.0037). (**B**) shows that the log capillary diameter at rest is significantly correlated with the log physical activities (r = 0.60; *p* = 0.0055). (**C**) shows that the log capillary flow at rest is significantly correlated with the log bone density (r = −0.80; *p* = 0.00001). (**D**) shows that the log capillary flow at rest is significantly correlated with the log bone density (r = 0.60; *p* = 0.0055). (**E**) shows that the log capillary density at rest is significantly correlated with the log bone density (r = 0.50; *p* = 0.024). (**F**) shows that the log capillary density at rest is significantly correlated with the log DBP (r = 0.46; *p* = 0.040). (**G**) shows that the log capillary diameter at rest is significantly correlated with the log BMI (r = −0.49; *p* = 0.029). (**H**) shows that the log age is significantly correlated with the log bone density (r = 0.50; *p* = 0.024).

All participants completed the experiments without any difficulty or side effects. The changes in pulse and blood pressure before and after exercise were safely managed, with pulse increases of no more than 30 bpm from resting rate, systolic blood pressure increases of no more than 40 mmHg, and diastolic blood pressure increases of no more than 20 mmHg. These parameters were in accordance with the exercise cessation criteria for hypertensive patients [24]. The participants’ characteristics are summarized in Table 1. As shown in Figure 4a, the capillary flow rate significantly changed from 2.3 ± 0.7 to 2.7 ± 0.2 log µm/s (*p* = 0.003), and the capillary density significantly changed from 0.8 ± 0.2 to 0.9 ± 0.1 log/mm (0.0003). However, no changes were observed in the diameter of the nailfold capillaries. Nailfold capillary images at baseline and after a 1 min exercise of a typical subject by the microscope measurement are shown in Figure 3b. In addition, the correlation of the nailfold capillary parameter at rest and other parameters is depicted in Figure 5: Figure 5A shows that the log capillary diameter at rest is negatively correlated with the log fat mass (r = −0.61; *p* = 0.0037); Figure 5B shows that the log capillary diameter at rest is correlated with the log physical activities (r = 0.60; *p* = 0.0055); Figure 5C shows that the log capillary flow at rest is negatively correlated with the log BMD (r = −0.8; *p* = 0.00001); Figure 5D shows that the log capillary flow at rest is correlated with the log physical activities (r = 0.60; *p* = 0.0055); Figure 5E shows that the log capillary density at rest is correlated with the log BMD (r = 0.50; *p* = 0.0243); Figure 5F shows that the log capillary density is correlated with the log DBP (r = 0.46; *p* = 0.040); Figure 5G shows that the log capillary density at rest is negatively correlated with the log BMI (r = −0.49; *p* = 0.0289); and Figure 5H shows that the log capillary diameter at rest is correlated with the log physical activities (r = 0.65; *p* = 0.029). Furthermore, the capillary diameter at rest was significantly multiple regressed with body fat mass and DBP. Moreover, in individuals who exhibited a change in the diameter of the nailfold capillary before and after exercise, a strong correlation of 0.75 was observed between the change in nailfold diameter (Δ nailfold diameter) and body fat mass (*p* = 0.012). Additionally, a moderate correlation of 0.64 was observed between age and systolic blood pressure (SBP) (*p* = 0.044).

On the other hand, in individuals who did not show a change or exhibited a decrease in the diameter before and after exercise, a strong negative correlation of −0.71 was observed between age and the change in flow of the nailfold capillary (Δ flow) (*p* = 0.021). A strong correlation of 0.95 was observed between the change in nailfold capillary flow (Δ nailfold flow) and physical activity (*p* = 0.000020). Furthermore, a strong negative correlation of -0.76 was observed between body fat mass and physical activities (*p* = 0.010).

## 4. Discussion

To the best of our knowledge, this is the first study to examine the immediate effects of exercise on capillaries and the safety of exercise for elderly individuals with hypertension. It also explored the correlation between capillary function and other health-related indicators. 

This study confirmed that all exercise protocols could be safely implemented, suggesting that even a 1 min exercise can safely and effectively enhance peripheral circulation, including in the non-exercising limb. Capillary examination parameters, now included in medical guidelines [25], can be improved through medication and lifestyle changes. Exercise and drug therapy have proven effective for dysfunctional vessels during recanalization [26]. However, excessive sympathetic nervous system responses to exercise may increase the risk of cardiovascular events, including circulatory dysfunction at movement onset, cardiac sympathetic overactivity, and orthostatic dysregulation [27,28].

The impact of local and acute exercises on capillaries remains unclear, highlighting the importance of investigating the acute effects of exercise on high-risk elderly individuals. Therefore, this study involved elderly participants over 60 years of age who do not exercise regularly. We measured the function of capillaries in the non-exercising limb before and after a 1 min moderate-intensity exercise. Significant changes were observed in the dysfunctional vessels of the non-exercising limb post-exercise. This could be attributed to two factors: First, the arm-curl exercise induced systemic circulation, despite contravening the specificity principle of exercise therapy. This is attributed to the effect on whole-body microcirculation rather than isolated skeletal muscle. Second, the capillary diameter remained unchanged, likely because the elderly hypertensive subjects had capillaries deformed into hairpin shapes with reduced function due to aging [29].

Capillaries, which are crucial for nutrient transport to cells, show a correlation with health-related indicators. Recent studies have reported an inverse relationship between body fat mass and capillary diameter, underscoring the importance of reducing body fat through continuous exercise therapy [30]. However, exercise therapy poses a risk of triggering cardiovascular events in high-risk elderly individuals [31], making sustained implementation challenging. This study found that moderate-intensity exercise was safe and effective, even for this high-risk group, demonstrating not only the acute effects of exercise but also long-term benefits such as improved capillary function, formation, and density [32], which emphasize the need to establish regular exercise habits.

The results also indicated that exercising healthy limbs in patients with hemiplegia or plaster fixation, which leads to inactivity, can positively influence the non-exercising limbs. Capillary function examinations could serve as an auxiliary method for evaluating treatment effectiveness, performed without significant increases in pulse or blood pressure changes, and at an intensity within a 13 RPE scale. Moreover, even short-term localized exercise can impact capillaries throughout the body, suggesting potential benefits for exercise prior to blood sampling or during dialysis treatment.

This study has several limitations: a small sample size, its designation as a pilot study, the absence of a control group, and the lack of analysis on long-term exercise effects or the comparative analysis of diseases and treatments in conditions such as diabetes and cerebrovascular diseases. As this study targeted elderly Japanese individuals, factors related to capillary function, including physique, diet, and physical activities, as well as lifestyle habits, may differ from those in Western and African countries [33]. Future studies should aim to increase the sample size, extend the intervention period, include comparisons of diseases, incorporate a control group, and conduct studies across different races.

## 5. Conclusions

In this study, community-dwelling elderly individuals with hypertension safely performed a 1 min exercise without experiencing serious adverse events, impacting whole-body capillary function. It was also suggested that capillary function is associated with health-related factors such as body fat and bone density. This approach can detect early microvascular changes associated with the risk in hypertensive elderly individuals and plays a significant role in the early prediction of whole-body function. Therefore, it may serve as a useful diagnostic tool for assessing nailfold capillary function.

## Figures and Tables

**Table 1:** Baseline characteristics of the participants.

Parameter	*n* = 20
Age (years)	67.2 ± 6.2
Male/female	7/13
Diabetes	4/20
BMI (kg/m^2^)	22.1 ± 3.6
Skeletal muscle mass (kg)	38.7 ± 6.2
Fat mass (kg)	22.6 ± 8.8
Systolic blood pressure (mmHg)	150.1 ± 17.9
Diastolic blood pressure (mmHg)	93.1 ± 11.7
CAVI	8.4 ± 1.0
ABI	1.1 ± 0.1
Diameter of the nailfold capillary	9.7 ± 2.6
Flow rate of the nailfold capillary	295.8 ± 172.2
Density of the nailfold capillary	7.0 ± 1.5
Bone mineral density (%)	26.3 ± 3.7 (n = 9)
Physical activities (steps/day)	4714.3 ± 1277.8 (n = 9)

Values represent the mean ± standard deviation of the 20 participants. BMI: body mass index, CAVI: cardio-ankle vascular index, ABI: ankle–brachial index.

## Data Availability

The data presented in this study are available on request from the corresponding author.
